# Technology and physical activity for preventing cognitive and physical decline in older adults: Protocol of a pilot RCT

**DOI:** 10.1371/journal.pone.0293340

**Published:** 2024-02-23

**Authors:** Savannah Kiah Hui Siew, Junhong Yu, Tat Lee Teo, Kuang Chua Chua, Rathi Mahendran, Iris Rawtaer

**Affiliations:** 1 Psychology, School of Social Sciences, Nanyang Technological University, Singapore, Singapore; 2 School of Engineering, Ngee Ann Polytechnic, Singapore, Singapore; 3 Yeo Boon Khim Mind Science Centre, National University of Singapore, Singapore, Singapore; 4 Department of Psychiatry, Sengkang General Hospital, Singapore, Singapore; University Hospital Cologne: Uniklinik Koln, GERMANY

## Abstract

**Background:**

Cognitive frailty, defined as having both physical frailty and cognitive impairment that does not satisfy the criteria for Major Neurocognitive Disorder, represents an elevated risk for morbidity. Hence, it is crucial to mitigate such risks. Physical activity interventions have been found effective in protecting against physical frailty and cognitive deterioration. This pilot RCT examines if smartwatches and mobile phone applications can help to increase physical activity, thereby improving physical and cognitive outcomes.

**Methods:**

Older individuals (n = 60) aged 60 to 85 years old will have their physical activity tracked using a smartwatch. The subjects will be randomized into two arms: one group will receive daily notification prompts if they did not reach the recommended levels of PA; the control group will not receive prompts. Outcome variables of physical activity level, neurocognitive scores, and physical frailty scores will be measured at baseline, T1 (3 months), and T2 (6 months). Sleep quality, levels of motivation, anxiety, and depression will be controlled for in our analyses. We hypothesize that the intervention group will have higher levels of physical activity resulting in improved cognitive and physical outcomes at follow-up. This study was approved by the National University of Singapore’s Institutional Review Board on 17 August 2020 (NUS-IRB Ref. No.: H-20-038).

**Discussion:**

Wearable sensors technology could prove useful by facilitating self-management in physical activity interventions. The findings of this study can justify the use of technology in physical activity as a preventive measure against cognitive frailty in older adults. This intervention also complements the rapidly rising use of technology, such as smartphones and wearable health devices, in our lives today.

**Registration details:**

This study has been retrospectively registered on clinicaltrials.gov on 5^th^ January 2021 (NCT Identifier: NCT04692974), after the first participant was recruited.

## Introduction

Cognitive and physical decline are two common ageing processes and they often interact. Physical frailty was found to be a risk factor for subsequent cognitive decline and mild cognitive impairment [1]. Similarly, cognitive impairment is an elevated risk for pre-frail and frail older adults [2, 3]. Thus, the term *cognitive frailty*, defined as having both physical frailty and cognitive impairment that does not satisfy the criteria for Major Neurocognitive Disorder [4], encapsulates both of these common ageing problems. In this paper, we are referring to this definition of cognitive frailty although it has recently been seen as a distinct concept within frailty which do not require the presence of physical frailty [5, 6]. A definition based on it being a distinct concept seems too similar to mild cognitive impairment to be useful. Diagnostic tools of cognitive frailty defer across studies and the resulting prevalence rates vary among community-dwelling populations from 2.7% to 12.2% [7–11]. Among these studies, populations in Asia showed lower prevalence rates of 2.7 to 6.3% [10, 11].

Adding the cognitive component to the definition of frailty has enhanced the predictive validity of adverse health outcomes [4]. Those with cognitive impairments face an increased prevalence of functional disability and mortality risks compared to those without cognitive impairment [12]. With cognitive frailty representing an elevated risk for morbidity, it is crucial to look into interventions that could help slow older adults’ arrival at this stage. One possible intervention is the promotion of physical activity. Physical activity is a main intervention for preventing and managing physical frailty. It was found to improve physical outcomes of pre-frail and frail older adults and maintain physical independence [13], even in as short as six weeks [14].

A wealth of research has been done regarding the role of physical activity on cognitive functioning. Low to moderate exercise had the effect of significantly protecting against cognitive decline in older adults without cognitive impairment [15, 16]. Similarly, meta-analysis results show that aerobic physical activity had an impact on the cognitive function of people with Alzheimer’s disease or dementia in general [17, 18]. These signals hope for the potential reversibility of cognitive decline. In recent years, our understanding of the mechanisms of physical activity on our well-being has increased. Possible factors that could play a role in the effects of physical activity are sleep quality, levels of motivation, and depression and anxiety [19]. We included these four factors to better understand the impact of physical activity.

With physical activity proving to be a potential method for delaying the onset or worsening of cognitive frailty, wearable sensors technology could represent a foray into such preventive intervention. Health-related usage of wearable sensors technology has found its way into mainstream markets with the development and adoption of smartwatches like the Apple Watch and Fitbits. Medical-grade wearable sensors have also been developed with more advanced technical capabilities. Most of the health research surrounding the use of these wearable sensors devices focuses on monitoring and tracking health-related signals. This includes stress monitoring [20], gait detection [21], monitoring of daily activities as an assistant to older’s people lives [22, 23], and making assessments on their fall risk [24, 25]. Wrist-worn wearable sensors have been validated against other gold standard measurements like skin conductance finger sensors and electrocardiography in measuring heart rate and heart rate variability [26]. The use of telehealth devices to encourage positive health behaviours has been studied more in recent times [27]. In one study, they found that pairing the Fitbit One with text message prompts could increase physical activity in the short term among participants who were of an unhealthily heavy weight range [28]. However, it is noted that even with the current pervasiveness of wearable sensors and mobile phones in our daily lives, the capabilities of such technology in healthcare interventions have yet to be widely validated [27]. Thus, the usability and efficacy of such technologies are still unclear.

## Methodology

### Experimental design

Our study aims to explore the use of wearable technology–in the form of smartwatches with notification prompts–in physical activity engagement on cognitive frailty outcomes. We postulate that wearable technology will help increase engagement in physical activity and improve cognitive and physical outcomes in older adults. The overarching goal is the prevention of cognitive frailty among this group of older adults.

This pilot study is a randomized controlled trial with a pre-test and post-test design. The independent variable explored in the study is the use of wearable technology and mobile application prompts while the levels of physical activity and physical and cognitive improvements are the dependent measures.

Data collection will be carried out at the research study site, by research assistants blinded to the participant’s group assignment. The active arm will receive notification prompts on their mobile applications if they did not reach the recommended levels of physical activity; the control arm will not receive prompts.

#### Recruitment procedure

Participants will be recruited from either an existing cohort study, the Community Health and Intergenerational (CHI) study, or via word-of-mouth referrals from sources such as senior activity centres or memory clinics. The CHI study is an ongoing cohort study investigating biopsychosocial factors of ageing among community-dwelling older adults aged 60 years old and above in Singapore [29]. Only subjects who gave their consent to be contacted for future research and met the inclusion criteria mentioned below will be invited to participate in the current study.

Research team members will obtain written informed consent from the participants before any study procedures and check that they meet the inclusion and exclusion criteria set out below in the “Participants” section before any study procedures.

#### Randomization procedure

Randomization will be carried out by a research assistant not involved in the study. Block randomization is used in this study to ensure that the two treatment arms have the same number of participants. Random numbers will be generated using block randomization (http://randomizer.org) with 30 sets of two numbers each. Each participant down the list will then be assigned to group 1 (intervention) or 2 (control) depending on the random numbers generated.

The assessors will be blind to the treatment assignment of the participant when administering the questionnaires. Group assignment and subsequent installation of the mobile phone application and smartwatch will only be done after completing baseline assessments.

### Participants

Sixty older adults aged 60 to 85 years old living in Singapore will be recruited via convenience sampling from the CHI study and word-of-mouth referral. If subjects currently engage in vigorous physical exercise, do not own an Android phone that can support at least a 6.0 Operating System, or have medical contraindications for physical exercise, they will be excluded from the study.

### Sample size justification

Given a hypothesized treatment effect size of 0.4, according to the meta-analysis of physical activity intervention on cognitive outcomes [30] and physical outcomes [31], it was estimated using G*Power [32] that a total sample size of 42 was required to detect a significant within-between interaction with a power of .80, assuming α = .05; the correlation between repeated measures = .50 and nonsphericity correction = 1. After considering potential drop-out rates of 20% and publication value, a total sample size of 60 was reached. This will be adequate to detect an effect size of 0.4 or larger, at a power of .80 if such an effect exists. In order to assess the feasibility of this RCT, recruitment and retention rates will be examined as secondary outcome measures.

### Material

#### Wearable sensors technology

The technology’s capability was developed in collaboration with Ngee Ann Polytechnic’s School of Engineering. The wearable device used is the Fossil Gen 4 Sports smartwatch, which allows tracking of the participant’s heart rate and the number of steps taken. Physical activity tracking is enabled on the watch to allow the collection of detailed information regarding physical exercise including type and duration of exercise, and heart rate information. Two language settings–English and Mandarin, will be available on the watch.

#### Mobile application

This will be linked to the smartwatches that participants wear. Participants have to own an Android phone which can support at least a version 6.0 Operating System. The application is meant to download and store information of interest from the smartwatches and encourage physical activity for those in the intervention group. The application will provide reminders in the form of push notifications if participants fail to reach their target for levels of physical activity. Two prompts–once at mid-day and once in the evening–will be sent out if target steps are not reached. Another prompt will be sent out in the middle of the week if target moderate-intensity exercises are not reached. The notification prompt aims to increase physical activity, and this is supplemented by information regarding workout classes, spaces, and facilities in their vicinity on the application. Safety messages regarding important information to note while exercising will be included in the mobile application as a reminder to participants to exercise carefully and safely. Similarly, two language settings–English and Mandarin, are available on the mobile application.

### Data collection

Baseline information on subjects’ demography, neurocognitive data, physical activity, and physical frailty will be collected. Follow-up assessments of both cognitive and physical outcomes will be done at 3 months and 6 months. The 6-month follow-up assessment allows us to test the sustainability of this intervention and rule out novelty effects. Secondary variables such as sleep quality, levels of motivation, anxiety, and depression will be included in the data collection. In all, participants have to make three visits and their entire participation will last six months.

#### Level of physical activity

Objective and subjective measurements of PA will be collected. The number of steps taken and heart rate data will be obtained from the smartwatches that the participants wear.

Subjective physical activity data will also be collected using the International Physical Activity Questionnaire short form (IPAQ) [33]. This collects information on hours of vigorous and moderate physical activity, walking, and sitting by the participant in the last seven days. This questionnaire was previously used in a study on physical activity amongst our local population [34]. A translated version of the IPAQ also showed adequate validity and reliability among a similar older adult population [35].

#### Outcomes measures

*Cognitive outcomes*. A neurocognitive assessment test battery (NCA) will be administered to measure cognitive outcomes. The NCA consisted of the following tests: Rey Auditory Verbal Learning Test (RAVLT), Digit Span—Forward and Backward, Colour Trails Test (CTT), Wechsler’s Block Design (WBD), and Semantic Verbal Fluency–Animals (SFA). RAVLT measures verbal and learning memory by getting participants to recall a list of words read aloud to them. Immediate and delayed recall and recognition tasks will be carried out. Working memory and attention will be assessed by the Digit Span task. Participants will listen to a string of numbers read aloud to them and recall them in the same order. They will subsequently do this in the backward order. CTT looks at sequencing and divided attention. Participants have to link up coloured circles on the task sheet in a particular order, as quickly as they can. WBD measures participants’ visuospatial ability where they have to arrange the blocks to form a given pattern. Lastly, SFA measures their verbal fluency. They are given one minute to list down as many animals as they can think of.

*Physical outcomes*. The FRAIL questionnaire [36] will be used. It is a simple questionnaire on Fatigue, Resistance, Ambulation, Illness, and Loss of weight. The scores range from 0 to 5 where each question is scored based on Yes (= 1) and No (= 0). FRAIL scores of 0 represent normal ageing, 1 to 2 represent pre-frail and 3 to 5 represent frail.

Although simple, the FRAIL scale has been validated to be a reliable screening test for frailty in many older adult community samples [36, 37] including our local population [38]. Often, the test can predict various adverse health consequences.

Additionally, physical performances related to frailty will be measured. These measurements, as recommended by the Asia-Pacific Clinical Practice Guidelines [39], include (1) Grip strength, (2) Gait speed, (3) Timed Up and Go (TUG), and (4) a portion of the Short Physical Performance Battery (SPPB). To prevent repeated measurements of similar components, only the time taken to rise from a chair and return to the seated position five times from the SPPB [40] will be measured.

#### Secondary outcomes

*Sleep quality*. Pittsburgh Sleep Quality Index (PSQI) [41] will be used as a subjective measure of the participant’s sleep quality. It is a self-report questionnaire intended to measure sleep quality over a one-month time frame. The questionnaire consists of 19 questions scored on a scale of 0 to 3 where 0 = not during the past month and 3 = three or more times a week. It measures seven different domains of sleep: (i) subjective sleep quality, (ii) sleep latency, (iii) sleep duration, (iv) habitual sleep efficiency, (v) sleep disturbances, (vi) use of sleep medication, and (vii) daytime dysfunction. Higher scores reflect poorer sleep quality and a global score of 5 or greater reflects poor sleep. The PSQI is a commonly used and validated tool with good reliability and validity [42]. However, when used on our local population, the scale’s reliability is relatively low (α = 0.62; α = 0.64–0.67) although still acceptable [43, 44].

*Self-efficacy*. The Barriers Self-Efficacy Scale (BARSE) [45] to measure physical-activity-specific self-efficacy will be used in this study. BARSE is the most commonly used scale among studies measuring self-efficacy concerning physical exercise interventions for Older Adults [46]. The scale consists of 13 questions where participants will rate their confidence to carry out certain behaviours in the presence of common barriers to exercising on a 10-point scale (1 to 100% in 10-point increments). The ratings are then summed up and averaged out by the number of questions to obtain a score for exercise-specific self-efficacy. Higher scores represent higher exercise-specific self-efficacy.

*Depression and anxiety*. The Geriatric Depression Scale (GDS) [47] and Geriatric Anxiety Inventory (GAI) [48] will be used to measure levels of anxiety and depression. GDS consists of 15 questions while GAI has 20 questions. Each question has a yes or no response with either a 0 or 1 score attached to it. The scores are then added up and a higher score represents higher levels of depression or anxiety symptoms. The GDS was established as a reliable and valid test among community-dwelling older adults in Singapore, with a Cronbach’s alpha of 0.80 and good inter-rater reliability and test-retest reliability [49]. GAI has shown good internal reliability among older adults of similar ethnicity, with Cronbach’s Alpha above 0.90 [50, 51] and significant concurrent validity with other measures of anxiety [51].

### Study procedure

#### Delivery of intervention

Participants in the intervention group will be given the smartwatch which they will use together with the accompanying mobile application for the period of the study. During the period of intervention, the watch will track the physical activity of the subject via the number of steps taken and the number of hours of moderate physical work (based on heart rate). Heart rate and steps will be tracked whenever participants are wearing the watch, which is when they are awake. The watch is charged nightly when they are sleeping. Participants will have to log down their physical activity by activating the physical activity tracker on the watch. If the subject did not hit the required level of physical activity, they will be sent a notification prompt through the mobile application and smartwatch. A map of nearby workout locations can also be accessed.

The physical activity guidelines for participants will be 150 minutes of moderate-intensity exercise in a week and 7500 steps daily. Most guidelines for exercising recommend a mix of different activities for older adults [52]. This includes both strength-based and aerobics physical activity [53]. As found in more recent review studies [17, 18], aerobic physical activity seems to be more advantageous. Thus, we rely on the intensity of the physical activity instead of the type. We do not intend to dictate the specific type of physical activity participants in our study engage in. As this study is primarily an efficacy exploration, we set a general guideline for the amount and level of physical activity instead and ask participants to clock in the type of exercise they did. Thereafter, we will use secondary analysis to look at whether the types of physical activity affect the results.

Effective physical activity dosage differs from person to person and the cognitive benefits are dependent on the individual’s response to the cardiorespiratory level of the activity [54]. Thus, using the participant’s heart rate to gauge the level of physical activity will help tailor the exercise dosage to that individual. Moderate intensity of physical activity is recommended [55]. Such activities will see a slight increase in breathing and heart rate and as a general rule of thumb, one should still be able to talk but not sing. The moderate intensity level can be determined by 50–70% of the participant’s Maximum Heart Rate (MHR). We help participants to calculate their MHR using the formula: 220 –Their age.

We are advocating for a 7500 steps goal because for our target demographic group, the healthy averages only 2000 to 9000 steps a day [56]. It is concluded that taking 7,100 steps a day is a reasonable goal for older adults that account for the recommended moderate-intensity exercise of 150 minutes a week. Moreover, over 7500 steps, mortality rates plateaued, showing no signs of further decrease and the intensity of steps taken had no clear association with mortality after considering the total step volume [57].

#### Control group

The control group will be told to carry on with their usual activities. They will also be given the same information on what the healthy physical activity guidelines are (i.e. 150mins of moderate intensity exercise a week and 7500 steps daily) during the first study visit. The control group will only wear the watch as a tracking device for us to obtain information regarding their heart rate and steps taken. They will not be able to see all of this information and their smartwatches are modified to only show them the time.

Additionally, no prompts or notifications will be given through the mobile phone application and it will be installed only for data collection purposes on our end.

### Participant timeline

**Fig 1:**
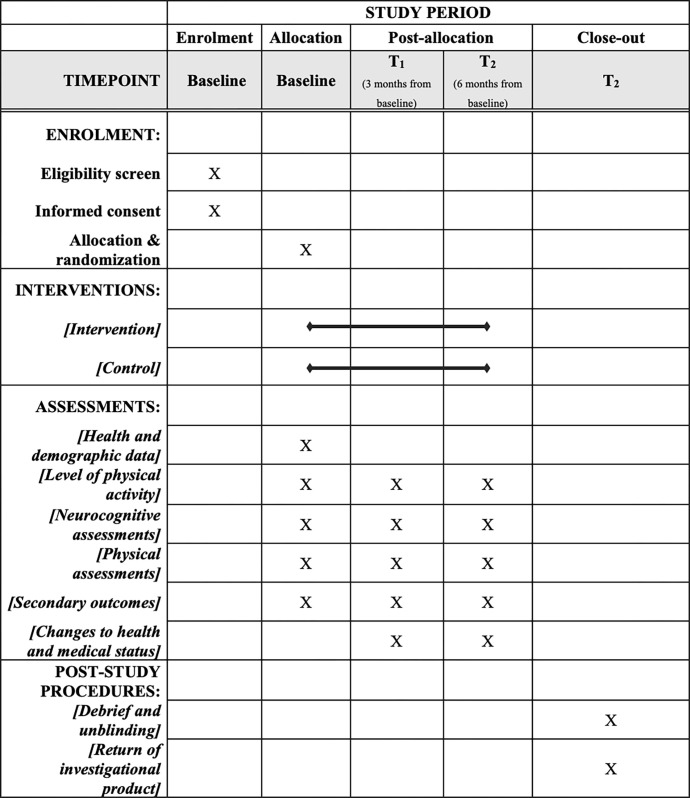
SPIRIT schedule of enrolment, interventions, and assessments diagram.

Fig 1 shows the enrolment, assessment and visit schedule for the study.

### Data management

Each subject will be coded with an identifier number at the first visit and the link between the code and the subject’s personal details will be kept separately. Documents with identifiers will also be password protected. Only study team members will have access to this information. Study team members involved with data collection will be properly trained and adequately monitored to ensure a good standard of data collection. Coded research data will be used for analysis. Personal data will not be used in a publication or presentation. All research data will be kept for a minimum of ten years after the completion of the study for regulatory and inspection purposes by authorized authorities.

Research assistants involved with data collection will be properly trained and adequately monitored to ensure a good standard of data collection. All data written on the assessment form or keyed into the online assessment form must not be erased or thrown away. If needed, cancellations must be accompanied by the signatures of the person making the changes. All paper trails must be kept and not thrown away for accounting purposes. Cross-checks of the data collated with the case report forms must be done during the process of the research study to ensure accuracy.

### Statistical analysis

The internal reliability of the scales used will be tested using Cronbach’s alpha. Outcome measures will be analyzed using generalized estimating equations with an AR1 working correlation structure. As long as participants have at least 1 timepoint of measurement, they will be included in the analyses. Sleep quality, levels of motivation, depression, and anxiety, will be controlled for in the analyse and effect size and confidence intervals calculations will be carried out. All statistical analyses will be carried out in R and finalized prior to conducting the analysis. Results will also be reported in accordance with the CONSORT reporting guidelines for randomised trials.

## Discussion

### Ethical and safety considerations

This study was approved by the National University of Singapore’s Institutional Review Board on 17 August 2020 (NUS-IRB Ref. No.: H-20-038). The conduct of the study will be guided by the Good Clinical Practice Principles and the Human Biomedical Research Act (2015) in Singapore. Written informed consent will be obtained from participants. An information sheet will be provided to them and they will be given sufficient time to consider their participation before being asked to sign the informed consent document. Additionally, the Mini-Mental State Examination (MMSE) with be administered to ensure that they have the cognitive capacity to give consent at the moment that consent is taken. The version of MMSE used has been modified for our local population with a set of locally validated norms adjusted for different education levels and ethnicity. For those with primary school education, the cutoff scores will be 19/20, and for those with secondary school and above education, the cutoff scores will be 22/23 [58]. Those who do not meet the MMSE cutoff scores, will not be invited to take part in the study.

After informed consent has been taken, participants will be screened for eligibility first. Subjects who do not meet the eligibility criteria will not be recruited for the study.

The risks involved in this study are expected to be minimal and no serious adverse effects are expected. Some mild fatigue may be experienced during the cognitive and physical assessments. Participants will be allowed to rest in between if required. The risk of overexertion during physical exercises is mitigated by ensuring recruited participants do not have any pre-existing conditions that preclude them from partaking in moderate exercise. Safety messages are also included in the mobile application to serve as a reminder. Moreover, exercises are intended to be of moderate intensity, and participants are not required to push to their limits. Any protocol amendments, deviations or adverse events will be reported to the ethics board and trial registries, if necessary.

### Dissemination plan

The study started recruitment in December 2020 and was expected to end in August 2021. However, due to restrictions and delays from the coronavirus pandemic situation causing multiple cessations of study procedures, the study has been extended to August 2023 and has now completed data collection. Findings from this study will be shared with the scientific community via peer-reviewed manuscript publications and conferences. A poster of the abstract of this protocol had been presented at the 20^th^ World Psychiatric Association’s World Congress of Psychiatry.

## Supporting information

S1 Checklist Spirit checklist. (DOC)

S1 Protocol Latest study protocol. (PDF)

## List of abbreviations

CHI: Community Health and Intergenerational study
IPAQ: International Physical Activity Questionnaire short form
NCA: Neurocognitive assessment test battery
RAVLT: Rey Auditory Verbal Learning Test
CTT: Colour Trails Test
WBD: Wechsler’s Block Design
SFA: Semantic Verbal Fluency–Animals
SPPB: Short Physical Performance Battery
PSQI: Pittsburgh Sleep Quality Index
BARSE: Barriers Self-Efficacy Scale
GDS: Geriatric Depression Scale
GAI: Geriatric Anxiety Inventory
ANOVA: Repeated-measures analysis of variance
MMSE: Mini-Mental State Examination

## References

[1] Boyle PA, Buchman AS, Wilson RS, Leurgans SE, Bennett DA. Physical frailty is associated with incident mild cognitive impairment in community-based older persons. Journal of the American Geriatrics Society. 2010;58(2):248–55. doi: 10.1111/j.1532-5415.2009.02671.x 20070417 PMC3150526

[2] Furtado GE, Caldo A, Rieping T, Filaire E, Hogervorst E, Teixeira AMB, et al. Physical frailty and cognitive status over-60 age populations: A systematic review with meta-analysis. Archives of Gerontology and Geriatrics. 2018;78:240–8. doi: 10.1016/j.archger.2018.07.004 30029093

[3] Panza F, Lozupone M, Solfrizzi V, Sardone R, Dibello V, Di Lena L, et al. Different Cognitive Frailty Models and Health-and Cognitive-related Outcomes in Older Age: From Epidemiology to Prevention. Journal of Alzheimer’s Disease. 2018;62(2018):993–1012. doi: 10.3233/JAD-170963 29562543 PMC5870024

[4] KelaiditiE, CesariM, CanevelliM, Abellan Van KanG, OussetPJ, Gillette-GuyonnetS, et al. Cognitive frailty: Rational and definition from an (I.A.N.A./I.A.G.G.) International Consensus Group. Journal of Nutrition, Health and Aging. 2013;17(9):726–34. doi: 10.1007/s12603-013-0367-2 24154642

[5] De Roeck EE, van der Vorst A, Engelborghs S, Zijlstra GAR, Dierckx E. Exploring Cognitive Frailty: Prevalence and Associations with Other Frailty Domains in Older People with Different Degrees of Cognitive Impairment. Gerontology. 2020;66(1):55–64. doi: 10.1159/000501168 31330515

[6] De Roeck EE, Dury S, De Witte N, De Donder L, Bjerke M, De Deyn PP, et al. CFAI-Plus: Adding cognitive frailty as a new domain to the comprehensive frailty assessment instrument. International Journal of Geriatric Psychiatry. 2018;33(7):941–7. doi: 10.1002/gps.4875 29637620

[7] Facal D, Campos-Magdaleno M, Navarro-Pardo E, Pereiro AX. Prevalence of cognitive frailty and associations with other frailty domains in a Spanish community-dwelling sample. Alzheimer’s & Dementia. 2020;16(S6):e041974.

[8] Navarro-Pardo E, Facal D, Campos-Magdaleno M, Pereiro AX, Juncos-Rabadán O. Prevalence of Cognitive Frailty, Do Psychosocial-Related Factors Matter? Brain Sciences. 2020;10(968):1–9. doi: 10.3390/brainsci10120968 33322251 PMC7763872

[9] RoppoloM, MulassoA, RabagliettiE. Cognitive frailty in Italian community-dwelling older adults: Prevalence rate and its association with disability. The journal of nutrition, health & aging. 2017;21(6):631–6. doi: 10.1007/s12603-016-0828-5 28537326

[10] RuanQ, XiaoF, GongK, ZhangW, ZhangM, RuanJ, et al. Prevalence of Cognitive Frailty Phenotypes and Associated Factors in a Community-Dwelling Elderly Population. The journal of nutrition, health & aging. 2020;24(2):172–80.10.1007/s12603-019-1286-732003407

[11] Shimada H, Makizako H, Doi T, Yoshida D, Tsutsumimoto K, Anan Y, et al. Combined Prevalence of Frailty and Mild Cognitive Impairment in a Population of Elderly Japanese People. Journal of the American Medical Directors Association. 2013;14(7):518–24. doi: 10.1016/j.jamda.2013.03.010 23669054

[12] Feng L, Zin Nyunt MS, Gao Q, Feng L, Yap KB, Ng TP. Cognitive Frailty and Adverse Health Outcomes: Findings From the Singapore Longitudinal Ageing Studies (SLAS). Journal of the American Medical Directors Association. 2017;18(3):252–8. doi: 10.1016/j.jamda.2016.09.015 27838339

[13] Kidd T, Mold F, Jones C, Ream E, Grosvenor W, Sund-Levander M, et al. What are the most effective interventions to improve physical performance in pre-frail and frail adults? A systematic review of randomised control trials. BMC Geriatrics. 2019;19(184):1–11. doi: 10.1186/s12877-019-1196-x 31291884 PMC6622112

[14] Losa-Reyna J, Baltasar-Fernandez I, Alcazar J, Navarro-Cruz R, Garcia-Garcia FJ, Alegre LM, et al. Effect of a short multicomponent exercise intervention focused on muscle power in frail and pre frail elderly: A pilot trial. Experimental Gerontology. 2019;115:114–21. doi: 10.1016/j.exger.2018.11.022 30528641

[15] Sofi F, Valecchi D, Bacci D, Abbate R, Gensini GF, Casini A, et al. Physical activity and risk of cognitive decline: A meta-analysis of prospective studies. Journal of Internal Medicine. 2011;269(1):107–17. doi: 10.1111/j.1365-2796.2010.02281.x 20831630

[16] Blondell SJ, Hammersley-Mather R, Veerman JL. Does physical activity prevent cognitive decline and dementia?: A systematic review and meta-analysis of longitudinal studies. BMC Public Health. 2014;14(510):1–12. doi: 10.1186/1471-2458-14-510 24885250 PMC4064273

[17] Groot C, Hooghiemstra AM, Raijmakers PGHM, van Berckel BNM, Scheltens P, Scherder EJA, et al. The effect of physical activity on cognitive function in patients with dementia: A meta-analysis of randomized control trials. Ageing Research Reviews. 2016;25:13–23. doi: 10.1016/j.arr.2015.11.005 26607411

[18] Jia RX, Liang JH, Xu Y, Wang YQ. Effects of physical activity and exercise on the cognitive function of patients with Alzheimer disease: A meta-analysis. BMC Geriatrics. 2019;19(181):1–14. doi: 10.1186/s12877-019-1175-2 31266451 PMC6604129

[19] Bherer L, Erickson KI, Liu-Ambrose T. A review of the effects of physical activity and exercise on cognitive and brain functions in older adults. Journal of Aging Research. 2013;2013(657508):1–8. 10.1155/2013/657508 PMC3786463 24102028

[20] Vos G, Trinh K, Sarnyai Z, Azghadi MR. Generalizable Machine Learning for Stress Monitoring from Wearable Devices: A Systematic Literature Review. International Journal of Medical Informatics. 2023:105026. doi: 10.1016/j.ijmedinf.2023.105026 36893657

[21] Prasanth H, Caban M, Keller U, Courtine G, Ijspeert A, Vallery H, et al. Wearable sensor-based real-time gait detection: A systematic review. Sensors. 2021;21(8):2727. doi: 10.3390/s21082727 33924403 PMC8069962

[22] Wang Y, Cang S, Yu H. A survey on wearable sensor modality centred human activity recognition in health care. Expert Systems with Applications. 2019;137:167–90.

[23] Rawtaer I, Mahendran R, Kua EH, Tan HP, Tan HX, Lee T-S, et al. Early detection of mild cognitive impairment with in-home sensors to monitor behavior patterns in community-dwelling senior citizens in Singapore: Cross-sectional feasibility study. Journal of medical Internet research. 2020;22(5):e16854. doi: 10.2196/16854 32369031 PMC7238076

[24] Chen M, Wang H, Yu L, Yeung EHK, Luo J, Tsui K-L, et al. A systematic review of wearable sensor-based technologies for fall risk assessment in older adults. Sensors. 2022;22(18):6752. doi: 10.3390/s22186752 36146103 PMC9504041

[25] Kristoffersson A, Du J, Ehn M. Performance and characteristics of wearable sensor systems discriminating and classifying older adults according to fall risk: a systematic review. Sensors. 2021;21(17):5863. doi: 10.3390/s21175863 34502755 PMC8434325

[26] Menghini L, Gianfranchi E, Cellini N, Patron E, Tagliabue M, Sarlo M. Stressing the accuracy: Wrist‐worn wearable sensor validation over different conditions. Psychophysiology. 2019;56(11):e13441. doi: 10.1111/psyp.13441 31332802

[27] Thilarajah S, Clark RA, Williams G. Wearable sensors and Mobile Health (mHealth) technologies to assess and promote physical activity in stroke: A narrative review. Brain Impairment. 2016;17(1):34–42.

[28] Wang JB, Cadmus-Bertram LA, Natarajan L, White MM, Madanat H, Nichols JF, et al. Wearable Sensor/Device (Fitbit One) and SMS Text-Messaging Prompts to Increase Physical Activity in Overweight and Obese Adults: A Randomized Controlled Trial. Telemedicine and e-Health. 2015;21(10):782–92. doi: 10.1089/tmj.2014.0176 26431257 PMC4842945

[29] Lee RZY, Yu J, Rawtaer I, Allen PF, Bao Z, Feng L, et al. CHI study: Protocol for an observational cohort study on ageing and mental health in community-dwelling older adults. BMJ Open. 2020;10(e035003):1–10. doi: 10.1136/bmjopen-2019-035003 32371513 PMC7229981

[30] Öhman H, Savikko N, Strandberg TE, Pitkälä KH. Effect of physical exercise on cognitive performance in older adults with mild cognitive impairment or dementia: A systematic review. Dementia and Geriatric Cognitive Disorders. 2014;38(5–6):347–65. doi: 10.1159/000365388 25171577

[31] De Labra C, Guimaraes-Pinheiro C, Maseda A, Lorenzo T, Millán-Calenti JC. Effects of physical exercise interventions in frail older adults: A systematic review of randomized controlled trials. BMC Geriatrics. 2015;15(154):1–16. doi: 10.1186/s12877-015-0155-4 26626157 PMC4667405

[32] Faul F, Erdfelder E, Buchner A, Lang AG. Statistical power analyses using G*Power 3.1: Tests for correlation and regression analyses. Behavior Research Methods. 2009;41(4):1149–60. doi: 10.3758/BRM.41.4.1149 19897823

[33] Craig CL, Marshall AL, Sjöström M, Bauman AE, Booth ML, Ainsworth BE, et al. International physical activity questionnaire: 12-Country reliability and validity. Medicine and Science in Sports and Exercise. 2003;35(8):1381–95. doi: 10.1249/01.MSS.0000078924.61453.FB 12900694

[34] Subramaniam M, Zhang Y, Lau JH, Vaingankar JA, Abdin E, Chong SA, et al. Patterns of physical activity and health-related quality of life amongst patients with multimorbidity in a multi-ethnic Asian population. BMC Public Health. 2019;19(1612):1–10. 31791301 10.1186/s12889-019-7941-4 PMC6889682

[35] Deng HB, Macfarlane DJ, Thomas GN, Lao XQ, Jiang CQ, Cheng KK, et al. Reliability and validity of the IPAQ-Chinese: The Guangzhou Biobank Cohort Study. Medicine and Science in Sports and Exercise. 2008;40(2):303–7. doi: 10.1249/mss.0b013e31815b0db5 18202571

[36] Morley JE, Malmstrom TK, Miller DK. A simple frailty questionnaire (FRAIL) predicts outcomes in middle aged african americans. Journal of Nutrition, Health and Aging. 2012;16(7):601–8. doi: 10.1007/s12603-012-0084-2 22836700 PMC4515112

[37] Woo J, Yu R, Wong M, Yeung F, Wong M, Lum C. Frailty screening in the community using the FRAIL scale. Journal of the American Medical Directors Association. 2015;16(5):412–9. doi: 10.1016/j.jamda.2015.01.087 25732832

[38] Merchant RA, Chen MZ, Tan LWL, Lim MYD, Ho HK, van Dam RM. Singapore Healthy Older People Everyday (HOPE) Study: Prevalence of Frailty and Associated Factors in Older Adults. Journal of the American Medical Directors Association. 2017;18(8):734.e9–.e14. doi: 10.1016/j.jamda.2017.04.020 28623152

[39] Dent E, Lien C, Lim WS, Wong WC, Wong CH, Ng TP, et al. The Asia-Pacific Clinical Practice Guidelines for the Management of Frailty. Journal of the American Medical Directors Association. 2017;18(7):564–75. doi: 10.1016/j.jamda.2017.04.018 28648901

[40] Guralnik JM, Simonsick EM, Ferrucci L, Glynn RJ, Berkman LF, Blazer DG, et al. A short physical performance battery assessing lower extremity function: Association with self-reported disability and prediction of mortality and nursing home admission. Journals of Gerontology. 1994;49(2):M85–94. doi: 10.1093/geronj/49.2.m85 8126356

[41] Buysse DJ, Reynolds CF III, Monk TH, Berman SR, Kupfer DJ. The Pittsburgh Sleep Quality Index: a new instrument for psychiatric practice and research. Psychiatry research. 1989;28(2):193–213. doi: 10.1016/0165-1781(89)90047-4 2748771

[42] Mollayeva T, Thurairajah P, Burton K, Mollayeva S, Shapiro CM, Colantonio A. The Pittsburgh sleep quality index as a screening tool for sleep dysfunction in clinical and non-clinical samples: A systematic review and meta-analysis. Sleep Medicine Reviews. 2016;25:52–73. doi: 10.1016/j.smrv.2015.01.009 26163057

[43] Koh HWL, Lim RBT, Chia KS, Lim WY. The Pittsburgh Sleep Quality Index in a multi-ethnic Asian population contains a three-factor structure. Sleep and Breathing. 2015;19(4):1147–54. doi: 10.1007/s11325-015-1130-1 25649251

[44] Dunleavy G, Bajpai R, Tonon AC, Chua AP, Cheung KL, Soh CK, et al. Examining the factor structure of the pittsburgh sleep quality index in a multi-ethnic working population in Singapore. International Journal of Environmental Research and Public Health. 2019;16(4590):1–12. 10.3390/ijerph16234590 PMC6926964 31756941

[45] McAuley E. The role of efficacy cognitions in the prediction of exercise behavior in middle-aged adults. Journal of Behavioral Medicine. 1992;15(1):65–88. doi: 10.1007/BF00848378 1583674

[46] French DP, Olander EK, Chisholm A, Mc Sharry J. Which Behaviour Change Techniques Are Most Effective at Increasing Older Adults’ Self-Efficacy and Physical Activity Behaviour? A Systematic Review. Annals of Behavioral Medicine. 2014;48(2):225–34. doi: 10.1007/s12160-014-9593-z 24648017

[47] Yesavage JA. Geriatric Depression Scale (Short Version). Psychopharmacology Bulletin. 1988;24(4):709–11. 3249773

[48] Byrne GJ, Pachana NA. Development and validation of a short form of the Geriatric Anxiety Inventory—The GAI-SF. International Psychogeriatrics. 2011;23(1):125–31. doi: 10.1017/S1041610210001237 20561386

[49] Nyunt MSZ, Fones C, Niti M, Ng TP. Criterion-based validity and reliability of the Geriatric Depression Screening Scale (GDS-15) in a large validation sample of community-living Asian older adults. Aging and Mental Health. 2009;13(3):376–82. doi: 10.1080/13607860902861027 19484601

[50] Yan Y, Xin T, Wang D, Tang D. Application of the Geriatric Anxiety Inventory-Chinese Version (GAI-CV) to older people in Beijing communities. International Psychogeriatrics. 2014;26(3):517–23. doi: 10.1017/S1041610213002007 24252312

[51] Dow B, Lin X, Pachana NA, Bryant C, Logiudice D, Goh AMY, et al. Reliability, concurrent validity, and cultural adaptation of the Geriatric Depression Scale and the Geriatric Anxiety Inventory for detecting depression and anxiety symptoms among older Chinese immigrants: An Australian study. International Psychogeriatrics. 2018;30(5):735–48. doi: 10.1017/S1041610217002332 29115201

[52] Freiberger E, Kemmler W, Siegrist M, Sieber C. Frailty and exercise interventions: Evidence and barriers for exercise programs. Zeitschrift fur Gerontologie und Geriatrie. 2016;49(7):606–11. 27655437 10.1007/s00391-016-1134-x

[53] Pavlova I, Vovkanych L, Vynogradskyi B. Physical activity of elderly people. Fizjoterapia. 2014;22(2):33–9.

[54] Vidoni ED, Johnson DK, Morris JK, Van Sciver A, Greer CS, Billinger SA, et al. Dose-response of aerobic exercise on cognition: A community-based, pilot randomized controlled trial. PLoS ONE. 2015;10(7):e0131647. doi: 10.1371/journal.pone.0131647 26158265 PMC4497726

[55] World Health Organization. Global recommendations on physical activity for health. Geneva: World Health Organization. 2010. 26180873

[56] Tudor-Locke C, Craig CL, Brown WJ, Clemes SA, De Cocker K, Giles-Corti B, et al. How many steps/day are enough? for adults. International Journal of Behavioral Nutrition and Physical Activity. 2011;8(79):1–17. doi: 10.1186/1479-5868-8-79 21798015 PMC3197470

[57] Lee I-M, Shiroma EJ, Kamada M, Bassett DR, Matthews CE, Buring JE. Association of step volume and intensity with all-cause mortality in older women. JAMA internal medicine. 2019;179(8):1105–12. doi: 10.1001/jamainternmed.2019.0899 31141585 PMC6547157

[58] Chua XY, Choo RWM, Ha NHL, Cheong CY, Wee SL, Yap PLK. Mapping modified Mini-Mental State Examination (MMSE) scores to dementia stages in a multi-ethnic Asian population. International Psychogeriatrics. 2018;31(1):147–51. doi: 10.1017/S1041610218000704 30017004

