# Supplementary material for: Technology and physical activity for preventing cognitive and physical decline in older adults: Protocol of a pilot RCT
Source: PLoS One. 2024 Feb 23;19(2):e0293340. doi: 10.1371/journal.pone.0293340 (PMC10889650; doi:10.1371/journal.pone.0293340)
Supplement: S1 Protocol — (PDF) [file pone.0293340.s002.pdf]

NUS Institutional Review Board (IRB)

IRB APPLICATION FORM

FOR HUMAN BIOMEDICAL RESEARCH (HBR)

I. BASIC INFORMATION

Protocol Title:

Cognitive Frailty in Older Adults: The role of technology in physical activity enhancement

Title	Name	Position	Dept./Institution
Dr			

Type of Study:

☐ Basic Research ☒ Clinical Research ☐ Database Review (non-medical records)

☐ Questionnaire / Survey / Interview / Focus Group ☐ Tissue Banking

☐ Emergency Research

☐ Others, please specify ____

Target Research Subjects are:

☐ Healthy Adults ☐ Minors (under 21) ☐ Pregnant Women ☐ Outpatients ☐ Prisoners

☒ Elderly ☐ Deceased

☐ Adults or minors who lack mental capacity - *please fill in section 4.2*.

☐ Minors who lack sufficient understanding and intelligence to give consent - *please fill in section 4.2*.

Research Subjects Will Be:

☒ Reimbursed $30 ☐ Not reimbursed ☐ Others - please specify: ____

Has this research been rejected by any IRB / REC / DERCs?

☒ No ☐ Yes If yes, please provide details: __________________

Study Site(s) ***(List all the site(s) of research, including PI's Dept & Institution)***

(NUS Sites) *(Please state all NUS depts if there is more than one)* :

OFFICIAL USE ONLY

(Non-NUS Sites) *(Please state the full address of premise(s) (including unit number & postal code):* Hannah Senior Activity Centre, Blk 21 Toh Yi Drive, #02-601 S(590021)

☒ Single-centre study – No. of NUS sites: 0

☐ Multi-centre study - No. of NUS sites:________

Total No. of Non-NUS sites: _____ (No. of local sites: _____ ; No. of overseas: ____)

If a Non-NUS site or external party is involved in the research, please indicate the applicable type of agreement with the external party:-

☐ Research Collaboration Agreement – Lead RI:________ Reviewing IRB:_________

☐ Service Agreement (*Please note NUS IRB will not review the service agreement, regardless whether NUS is provider or recipient of the service. PI is advised to ensure the requisite ethics and other regulatory approvals are covered contractually)*

This research is also submitted to or approved by:

☐ NHG Domain-Specific Review Board (DSRB) A / B / C / D / E / F

☐ SingHealth Centralized IRB (CIRB) A / B / C / D / E / F

☐ Others, please specify __________

☒ **Not Applicable**

HBR Checklist

This Research Involves:

(N.B. If none of the boxes here are ticked, you are advised to use the SBER forms for your application)

Study Objectives/Processes:

☒ The prevention, prognostication, diagnosis or alleviation of any disease, disorder or injury affecting the human body.

☐ The restoration, maintenance or promotion of the aesthetic appearance of human individuals through clinical procedures or techniques.

☐ The performance or endurance of human individuals.

☐ The research involves any of the following:

- ☐Human gametes or human embryos.
- ☐Cytoplasmic hybrid embryos.
- ☐Introduction of any human-animal combination embryo into an animal or a human, or any entity created as a result of this process.

☐Introduction of human stem cells (including induced pluripotent stem cells) or human neural cells into an animal at any stage of development (including a prenatal animal foetus or animal embryo), or any entity created as a result of this process.

Intervention: *(including any willful act or omission, that has a physical, mental or physiological effect (whether temporary or permanent) on the body of the individual)*

☒ Yes ☐ No

Biological Samples:

☐ Human biological materials (excluding human tissues) – identifiable / coded.

☐ Human biological materials (excluding human tissues) – anonymised.

☐ Human tissues – identifiable / coded.

☐ Human tissues – anonymised.

Health Information:

☒ Health information (not obtained from medical records) – identifiable / coded.

☐ Health information (not obtained from medical records) – anonymised.

☐ Medical records/clinical data from healthcare institutions, e.g. clinics and hospitals (including MRI, CT scans, blood test and etc.).

Please specify source: ____

The records are: ☐ Identifiable / coded to the research team.

☐ Anonymised to the research team.

II. DECLARATION OF THE PRINCIPAL INVESTIGATOR		
I hereby declare that:		
**Prohibited and Restricted Research**	**Yes**	**No**
a. I am not conducting any prohibited human biomedical research as defined in the Third Schedule of the Human Biomedical Research Act (HBRA).	☒	☐
b. I am not conducting restricted human biomedical research as defined in the Fourth Schedule of the HBRA.	☒	☐
c. I am conducting restricted human biomedical research as defined in the Fourth Schedule of the HBRA and will comply with the HBRA and its Regulations AND all conditions imposed by the MOH, including but not limited to the punctual submission of periodic reports at such times and intervals as may be determined by MOH.	☐	☒
**Consent**	**Yes**	**No**
d. I will ensure that appropriate consent has been obtained from the research subjects in accordance with Part 3 of the HBRA prior to:<br>i. The participation of the research subject, and/or<br>ii. The use of individually-identifiable biological materials, and/or<br>iii. The use of individually-identifiable health information of the research subject and/or<br>iv. The removal, donation or use of human tissue.	☒	☐
e. If conducting prospective recruitment, I will use an IRB-approved participant information sheet and consent form to obtain consent from subjects.	☒	☐
**Privacy and Confidentiality**	**Yes**	**No**
f. Research data will be retained in accordance to NUS' Research Data Management Policy.	☒	☐
g. I will protect research subjects' privacy and the confidentiality of their personal data, and comply with all relevant laws and regulations, institutional guidelines and policies including the Research Compliance Policy on Human Subject Research and Human Biomedical Research and the NUS Data Protection Policy.	☒	☐
**Additional Declarations, if applicable**	**Yes**	**NA**
h. I will obtain IACUC approval if animals are used in this research.	☐	☒
i. I confirm that my research is not regulated as a "clinical trial" under the Health Products (Clinical Trials) Regulations or the Medicines (Clinical Trials) Regulations.	☐	☒
j. For unregistered medical device that is imported, or locally manufactured and supplied, for use in my research, I confirm that I have submitted a Clinical Research Material (CRM) Notification to Health Sciences Authority (HSA).	☐	☒
**Final Declarations**	**Yes**	**No**
k. I confirm that my research team is fully qualified and properly trained to conduct the proposed research herein. To this end, I have submitted the updated CVs of myself and the research team for the IRB's review.	☒	☐
l. This research has not commenced and I will not initiate this research until I receive written notification of NUS-IRB approval and any other required approval(s) from relevant authorities.	☒	☐

m. I will not deviate from the IRB-approved protocol without prior written approval from NUS-IRB except unless it is necessary to mitigate an immediate risk of harm to the research subjects. Any protocol deviation will be reported to the NUS-IRB without unreasonable delay.	☒	☐
n. I will maintain all relevant documents and recognize that the NUS-IRB staff, Research Compliance and Integrity Office staff and regulatory authorities may inspect these records.	☒	☐
o. I have assessed the suitability of the premise(s) for the proposed research and will obtain NUS Office of Safety Health and Environment (OSHE) and other relevant approval(s), if required.	☒	☐
p. I understand that failure to comply with all applicable regulations, research compliance, institutional and NUS-IRB policies and requirements may result in the suspension or termination of this research, and other actions as stated in the NUS Code & Procedures on Research Integrity.	☒	☐
q. I will promptly report all serious adverse events, unanticipated problems or incidents, or contraventions of the HBRA that may occur in the course of this research in accordance with the Research Compliance Policy on Human Subject Research and Human Biomedical Research.	☒	☐
r. I declare that there is no existing or potential conflict of interest for any of the investigators participating in this research and/or their immediate family members. If there are conflicts of interest, I have declared them accordingly.	☒	☐

***Please state the name and email address of the person(s) to copy to in our acknowledgement email. If no name(s) is listed, the IRB Secretariat will only email the PI.**

1. Savannah Siew (pcmskhs@nus.edu.sg)

Note: ***Electronic signatures will suffice.***

IV. COMMENTS OF THE HEAD OF DEPARTMENT *

(Please check the appropriate box. Indicate "NA" if not applicable.)

	YES	NO
**1. Significance:**		
Does the research address an important scientific problem? Will the research affect concepts and methods that drive the field?	☐	☐
**2. Approach:**		
Is the conceptual framework adequately developed? Are the design, methods and analyses adequately developed and appropriate?	☐	☐
**3. Innovation:**		
Does the research challenge existing paradigms? Does it employ novel concepts, approaches and methods?	☐	☐
**4. Principal Investigator:**		
Is the Principal Investigator qualified and suitable to conduct this research? Does the Principal Investigator have evidence of commitment (e.g. previous track record)?	☐	☐
**5. Environment:**		
Is the Principal Investigator's environment suitable for the conduct of the proposed research?	☐	☐
**6. Budget (if study is funded):**		
Are the projected costs appropriate (i.e. accurate)? Is the overall budget reasonable for the significance of the research?	☐	☐

Comments

____

____

____

____

____

** IMPORTANT - Please complete ALL sections.

V. ABSTRACT OF RESEARCH PROPOSAL

In no more than 300 words, *describe concisely the specific aims, hypotheses, methodology and approach of the application, indicating where appropriate the application's importance to science or medicine. The abstract must be self-contained so that it can serve as a succinct and accurate description of the application when separated from it. Please use lay terms and spell out all acronyms when they are used the first time. If it is not possible to use lay terms, the technical and medical terms should be explained in simple language.*

Our study aims to explore the role of technology -- in the form of smart watches and mobile phone application -- in physical activity enhancement on cognitive frailty outcomes. Cognitive frailty is defined here as having both physical frailty and cognitive impairment but does not satisfy criteria for Major Neurocognitive Disorder. We postulate that for pre-frail older adults with mild cognitive impairment, such technology will help increase engagement in physical activity with subsequent improvement in cognitive and physical outcomes at follow up. This is with the aim of preventing this particular group from deteriorating to cognitive frailty because of the accompanying increased risk for adverse outcomes and morbidity.

This pilot study will be a randomized control trial with 2 treatment arms. Assessments will be done prior to and following the intervention period. During the period of intervention, the wearable will act as a tracking device and will be paired with a mobile application to issue prompts to the participant when necessary. The independent variable explored in the study is the use of the wearable while the levels of physical and cognitive improvements are the dependent measures. These will be tracked at baseline, 3 months and 6 months. Additionally, the mediating variable measured is the levels of physical activity to ensure that the proposed outcomes are affected through an increased level of physical activity encouraged by the use of the device.

If innovations like technology and the role of self-management proves efficacious, the future of healthcare in the context of a rapidly aging population will be more sustainable. Furthermore, this supporting role of technology in positive behavioral modification amongst older adults can have a multitude of applications in subsequent healthcare interventions.

VI. PROTOCOL DETAILS

Organise details of the research protocol under the following headings (in no more than 7 pages).

1. Specific Aims:

1.1 State concisely and realistically the purpose of the biomedical research, what the research described in this application is intended to accomplish and/or what hypothesis is to be tested.

This study intends to determine if smart watches and mobile phone application prompts can complement physical activity as a preventive intervention by motivating them to exercise, so as to improve their physical and cognitive outcomes.

We hypothesize that technology will help increase engagement in physical activity for the intervention group relative to the control group and subsequently improve cognitive and physical outcomes.

2. Introduction:

2.1 Briefly describe the background and the importance of the research.

The future of healthcare for older adults poses a unique problem for Singapore as our space constraints limits our ability in dealing with this issue and as Singapore's aging population is one of the fastest growing in Asia. Turning to technology and self-management seems necessary and research has to gather pace to look into the efficacy of programs with such aspects.

Physical frailty often interacts with cognitive decline (Boyle, et al., 2010; Furtado, et al., 2018; Panza, et.al., 2018) amongst this population. The term cognitive frailty was conceptualised to reconcile the associations (Furtado, et.al., 2018) and overlaps that the 2 concepts, Dementia and physical frailty had. It is defined as having both physical frailty and cognitive impairment that does not satisfy criteria for Major Neurocognitive Disorder (Kelaiditi, et al., 2013).

Adding the cognitive component to the definition of frailty have enhanced the predictive validity of adverse health outcomes (Kelaiditi, et. al., 2013) such as an increased prevalent rate of functional disability and mortality risks (Feng, et. al., 2017). With cognitive frailty representing an elevated risk for morbidity, it is crucial to look into interventions that could help slow older adult's arrival at this stage. One possible intervention is the promotion of physical exercise — which has been found effective in preventing and protecting against physical frailty and cognitive deterioration (Blondell et al., 2014; Kidd, et al., 2019; Losa-Reyna, et al., 2019; Sofi, et. al., 2010). There are also promising findings of the potential reversibility of cognitive decline. RCT studies show that aerobic physical activity had an impact on cognitive function among people with Alzheimer's Disease (Jia, et al., 2019) or Dementia in general (Groot, et al., 2016).

With increasing physical activity proving to be a potential method for delaying the onset or worsening of cognitive frailty, technology such as smart watches and mobile phone applications could represent a foray into such preventive intervention. Using tele-health devices to encourage positive health behaviours have been experimented with more in recent times (Thilarajah, et al., 2016; Wang, et. al., 2015). The step towards self-management and technology can complement and ease the burden on the future healthcare system. However, even with the current pervasiveness of wearable sensors and mobile phones in our daily lives, capabilities of such technology in healthcare interventions have yet to be widely validated. Thus, the usability and efficacy of such technologies are still unclear.

2.2 Relevant references

1. Blondell, S. J., Hammersley-Mather, R. & Veerman, J. L. (2014). Does physical activity prevent cognitive decline and dementia?: A systematic review and meta-analysis of longitudinal studies. *BMC Public Health, 14*(510), 1-12. DOI: 10.1186/1471-2458-14-510
2. Boyle, P. A., Buchman, A. S., Wilson, R. S., Leurgans, S. E. & Bennett, D. A. (2010). Physical frailty is associated with incident mild cognitive impairment in community-based older persons. *Journal of the American Geriatrics Society, 58*(2), 248-255. DOI: 10.1111/j.1532-5415.2009.0267.x
3. Feng, L., Nyunt, M. S. Z., Gao, Q., Feng, L., Yap, K. B. & Ng, T.-P. (2017). Cognitive frailty and adverse health outcomes: Findings from the Singapore Longitudinal Ageing Studies (SLAS). *Journal of Post-Acute and Long-Term Care Medicine*, *18*(1), 252-258. DOI: 10.1016/j.jamda.2016.09.015
4. Furtado, G. E., Caldo, A., Rieping, T., Filaire, E., Hogervorst, E., Teixeira, A. M. B. & Ferreira, J. P. (2018). Physical frailty and cognitive status over-60 age populations: A systematic review with meta-analysis. *Archives of Gerontology and Geriatrics*, *78*(1), 240-248. DOI: 10.1016/j.archger.2018.07.004
5. Groot, C., Hooghiemstra, A. M., Raijmakers, P. G. H. M., van Berckel, B. N. M., Scheltens, P., Scherder, E. J. A, van der Flier, W. M. & Ossenkoppele, R. (2016). The effect of physical activity on cognitive function in patients with dementia: A meta-analysis of randomized control trials. Ageing Research Reviews, 25(1), 13 – 23. https://doi.org/10.1016/j.arr.2015.11.005
6. Jia, R., Liang, J., Xu, Y. & Wang, Y. (2019). Effects of physical activity and exercise on the cognitive function of patients with Alzheimer disease: A meta-analysis. BMC Geriatrics, 19(181), 1 – 14. https://doi.org/10.1186/s12877-019-1175-2
7. Kelaiditi, E., Cesari, M., Canevelli, M., Abellan Van Kan, G., Ousset, P.-J., Gillette-Guyonnet, S., Ritz, P., Duveau, F., Soto, M. E., Provencher, V., Nourhashemi, F., Salvà, A., Robert. P., Andrieu, S., Rolland Y., Touchon, J., Fitten, J. L. & Vellas, B. (2013). Cognitive frailty: Rational and definition from an (I.A.N.A/I.A.G.G.) international consensus group. *The Journal of Nutrition, Health and Aging, 17*(9), 726-734.
8. Kidd, T., Mold, F., Jones, C., Ream, E., Grosvenor, W., Sund-Levander, M., Tingström, P. & Carey, N. (2019) What are the most effective interventions to improve physical performance in pre-frail and frail adults? A systematic review of randomized control trials. *BMC Geriatrics, 19*(184), 1-11. DOI: 10.1186/s12877-019-1196-x
9. Losa-Reyna, J., Baltasar-Fernandez, I., Alcazar, J., Navarro-Cruz, R., Garcia-Garcia, F. J., Alegre, L. M. & Alfaro-Acha, A. (2019) Effect of a short multicomponent exercise intervention focused on muscle power in frail and pre-frail elder: A pilot trial. *Experimental Gerontology, 115*(1), 114-121. DOI: 10.1016/j.exger.2018.11.022
10. Panza, F., Lozupone, M., Solfrizzi, V., Sardone, R., Dibello, V., Di Lena, L., D'Urso, F., Stallone, R., Petruzzi, M., Giannelli, G., Quaranta, N., Belloma, A., Greco, A., Daniele, A., Seripa, D. & Logroscino, G. (2018). Different cognitive frailty models and health- and cognitive-related outcomes in older age: From epidemiology to prevention. *Journal of Alzheimer's Disease, 62*(1), 993-1012. DOI: 10.3233/JAD-170963
11. Sofi, F., Valecchi, D., Bacci, D., Abbate, R., Gensini, G. F., Casini, A. & Macchi, C. (2010). Physical activity and risk of cognitive decline: A meta-analysis of prospective studies. *Journal of Internal Medicine, 269*(1), 107-117. DOI: 10.111/j.1365-2796.2010.02281.x

12. Thilarajah, S., Clark, R. A. & Williams, G. (2016). Wearable sensors and mobile health (mHealth) technologies to assess and promote physical activity in stroke: A narrative review. Brain Impairment, 17(1), 34-42. DOI: 10.1017/Brlmp.2016.1
13. Wang, J. B., Cadmus-Bertram, L. A., Natarajan, L., White, M. M., Madanat, H., Nichols, J. F., Ayala, G. X. & Pierce, J. P. (2015). Wearable sensor/device (Fitbit One) and SMS text-messaging prompts to increase physical activity in overweight and obese adults: A randomized controlled trial. *Telemedicine and e-HEALTH*, *21*(10), 782-792. DOI: 10.1089/TMC.2014.0176

3. Preliminary Studies:

3.1 Provide a brief account of the Principal Investigator's preliminary/pilot studies (if any) pertinent to the application. Please note that the IRB approval granted for this study will not cover preliminary/pilot studies that have already been conducted or are ongoing.

This research is intended to be a feasibility study into the effectiveness of technology such as smart watches and mobile phone application in the lives and well-being of older adults. No preliminary/pilot studies were done prior to this.

However, participants will be recruited from an ongoing elderly cohort study (Community Health and Intergenerational study) also under the Principal Investigator of this study. That study is collecting biological, psychological and social health determinants of community-dwelling elderly in Singapore.

4. Methodology:

4.1 Describe in detail the (i) experimental design and research procedures, (ii) research visits by the research subject (frequency and duration of procedures involved) and (iii) period of recruitment to accomplish the aims of this research.

(i) This pilot study will be a randomized control trial with pre and post testing. Participants will either be randomly assigned to the intervention group or to the control group. The independent variable explored in the study is the use of the wearable technology and mobile application prompts while the levels of physical and cognitive improvements are the dependent measures. Additionally, the mediating variable measured is the levels of physical activity to ensure that proposed outcome measures are affected through an increased level of physical activity encouraged by the use of the device.

60 older adults aged 60 to 85 years old who are pre-frail with MCI will be recruited for this study. They will be recruited from an ongoing elderly cohort study, the Community Health and Intergenerational (CHI) study at the NUS Mind-Science Center. Since our study intends to encourage physical activity through wearable technology and mobile applications among those who have low activity and are at risk for cognitive and physical decline, older adults who are physically very active or have medical contraindications will be excluded.

Participants in the intervention group will be given the wearables which they will use together with the accompanying mobile application for the period of the study. During the period of intervention, the wearable will track the physical

activity of the older adults via the number of steps taken and number of hours of moderate physical work (based on heart rate). Heart rate and steps will be tracked whenever participants are wearing the watch, which is when they are awake. The watch is to be charged every night when they are sleeping. Participants will have to log down their physical activity by activating the physical activity tracker either on the watch or on the mobile application. If they did not hit the required level of physical activity, they will be sent a notification prompt through the mobile application with details of nearby workout locations as recommendation. For the control group, they will wear the wearables as a tracking device. No prompts will be given and the mobile application will only be installed but not used for this group.

The physical activity guidelines for participants will be 150 minutes of moderate intensity exercise in a week and 7500 steps daily. The technology's capability was developed in collaboration with students from Ngee Ann Polytechnic's School of Engineering. The wearable smart watch used will be the Fossil Smartwatch Sloan HR which enables tracking of the participant's heart rate and steps taken. Those in the intervention group will be able to view this information. However, the control group will not be able to obtain these information and their smart watches will be modified to only show them the time. The mobile application will be linked to the smart watches that participants wear. Participants have to own an Android phone which can support at least a version 6.0 Operating System. The application is meant to be a form of tracking and encouragement of physical activity for those in the intervention group. It will provide reminders in the form of push notifications if participants fail to reach their target for levels of physical activity. Two prompts – once at mid-day and once in the evening – will be sent out if target steps are not reached. Another prompt will be sent out in the middle of the week if target moderate intensity exercises are not reached. Those in the control group will not be receiving the reminders and notifications from the mobile application.

Baseline information on their personal demographics, neurocognitive data, physical activity and physical frailty will be collected. Level of physical activity is measured using objective data from the smart watch and the International Physical Activity Questionnaire (IPAQ) which collects information on hours of vigorous and moderate physical activity, walking and sitting by the participant in the last 7 days. The FRAIL questionnaire, a simple screening test for frailty in many elderly community samples, hand grip strength, Gait speed, Timed Up and Go (TUG) and a portion of the Short Physical Performance Battery (SPPB) will be used to measure physical frailty. A test battery of NCA that was used in the CHI study will be administered. This includes various commonly used measures of cognition: Rey Auditory Verbal Learning Test (RAVLT), Digit Span Forward and Backward (DS), Colour Trails Test (CTT), Wechsler's Block Design (WBS) and Semantic Verbal Fluency – Animals (SFA). In the event that social distancing measures due to COVID-19 are still in place, the Cambridge Neuropsychological Test Automated Battery (CANTAB) will be used to assess cognitive outcome instead. It is a computerized cognitive test, useful for tracking cognitive changes over time.

Follow up assessments of both cognitive and physical outcomes will be done at 3 months and 6 months. Follow up assessments allow us to test the sustainability of this intervention and to rule out novelty effects. Secondary variables such as sleep quality, levels of motivation, anxiety and depression will be included in the data collection. The questionnaires used are Pittsburgh Sleep Quality Index (PSQI), Barriers Self-Efficacy Scale (BARSE), Geriatric Depression Scale (GDS) and Geriatric Anxiety Inventory (GAI).

(ii) In all, participants will have to make 3 research visits and their entire participation will last 6 months.

	Procedures	Duration

Visit 1 (T_1)	1. Treatment allocation and giving out research materials (smart watch, instructions, downloading of mobile phone application)<br>2. Personal demographics and related questions<br>3. NCA<br>4. Physical frailty measures<br>5. IPAQ<br>6. Secondary measures (PSQI, BARSE, GDS, GAI)	2 hours
Visit 2<br>(T_2 = 3 months)	1. NCA<br>2. Physical frailty measures<br>3. IPAQ<br>4. Secondary measures (PSQI, BARSE, GDS, GAI)	1.5 hours
Visit 3<br>(T_3 = 6 months)	1. NCA<br>2. Physical frailty measures<br>3. IPAQ<br>4. Secondary measures (PSQI, BARSE, GDS, GAI)<br>5. Return the smart watches	1.5 hours

(iii) Subjects will be recruited over a period of 4 months.

4.2 Are you recruiting adults or minors who lack mental capacity, or minors who lack sufficient understanding and intelligence to give consent? ☐ Yes, please specify: _____ ☒ No

If Yes, please explain why there are reasonable grounds for believing that biomedical research of comparable effectiveness cannot be carried out without the participation of the class of persons to which the adult/minor belongs.

4.3 Include details on sample size calculation and the means by which data will be analysed and interpreted.

Given a hypothesized treatment effect size of 0.4 according to previous similar studies, it was estimated using G*Power that a total sample size of 42 was require to detect a significant within-between interaction with a power of .80 (assuming a = .05; correlation between repeated measures = .50; nonsphericity correction = 1). After considering for potential drop-out rates and publication value, a total sample size of N=60 was reached. Given that our valid sample size (N = 60) is larger than 42, our sample size is more than adequate to detect an effect size of 0.4 or larger, at a power of .80 if such effect exists.

The intervention outcomes will be analysed using repeated measures analysis of variance (ANOVA) and linear mixed models.

4.4 What are the reasonably foreseeable risks, discomforts or inconveniences to a living research subject arising from this biomedical research and/or removal of tissue? Please also

state the expected serious / adverse events that may occur in this research. Please state the measures that will be in place to mitigate any risk of such harm to the research subject.

No serious adverse effects from the research are expected and risks are minimal.

During the cognitive assessments, subjects may experience mild fatigue since they are required to concentrate on the task at hand. They will be allowed to rest in between assessments if require.

During the physical exercises, participants may also experience fatigue and might be at risk of over-exerting themselves. This risk is mitigated by ensuring that participants do not have any existing conditions that precludes them from partaking in moderate exercise. We will also include safety messages in the accompanying mobile application on safety while exercising. Moreover, exercises are intended to be of moderate intensity and participants are not required to push to their limits.

4.5 What are the benefits that the research subject may reasonably expect from the biomedical research? Note that reimbursements to research subjects are not construed as benefits of the research.

Research subjects in the intervention group can reap the health benefits of exercising and will be able to track their activity levels via the wearable sensor. Known health benefits of physical exercise includes reduced risk of obesity and heart diseases. It may also improve physical frailty and cognitive abilities.

4.6 Are there any alternative procedures or treatments available to the research subject if they do not participate in this research? If so, what are the potential benefits and risks of such alternatives?

As MCI and pre-frail are not medical conditions and there are no recommended treatments beyond behavioural interventions such as exercise and proper diet, no alternative procedures or treatments will be available to the research subject if they do not participate in this research.

4.7 What compensation and treatment are available to the research subject in the event of injury arising from participation in the research and/or donating tissue and who will be responsible to pay for research related injuries, when arise?

[Note: With effect from 1 Jan 2016, NUS has put in place an annual blanket Clinical Trial Insurance Policy for all NUS-IRB approved research studies conducted in NUS. Please refer to https://staffportal.nus.edu.sg/staffportal/finance/insurance/summary-of-policy-cover.html for more details.]

Any research-related injuries will be covered by the NUS Clinical Trial Insurance Policy.

4.8 *What are the anticipated expenses the research subject is likely to incur as a consequence of participating in the biomedical research and/or donating tissue?*

No expenses are expected from the research subjects.

4.9 *Do you anticipate any incidental findings in this research? If so, please state your plan for management of incidental findings. Please note that NUS adopts the policy of granting the research subject the choice as to whether they wish to be identified and informed in the event of an incidental finding if the research expressly provides for such re-identification. Please refer to the NUS Management of Incidental Findings Policy (https://staffportal.nus.edu.sg/staffportal/research/research-compliance-integrity/).*

We do not anticipate any incidental findings in this research.

4.10 *Will any part of the procedures be audio-recorded, video-recorded, or placed on other electronic media?* ☐ *Yes, please specify:* ______ ☒ *No*

If Yes, explain how the recorded information will be used? Will information collected be published with or without identifying the research subjects? How long will the recordings be retained and how will they be disposed of? NA

Will research subjects who decline recording be excluded from the study? ☐ *Yes* ☐ *No, please specify alternative:* NA

5. Additional Information on Methodology:

☐ *5.1 Please fill in the following information on measures you will take to protect the research data and personal data collected. In addition, if your research involves making use of archived/ existing databases, please furnish the necessary documentation, e.g., permissions to use those databases, if applicable:*

5.1.1 *Please state whether the participation of the research subjects involves information in individually-identifiable form and the personal data that will be collected (e.g., names and contact information, etc.).*

For the purposes of contacting subjects during the research, names and contact information will be collected and kept separately from the research data and under lock and key.

5.1.2 *Please state the extent to which information identifying the research subject will be kept confidential and how research subjects' privacy will be protected. What will happen to the personal data collected after completion of the research study?*

Each subject will be coded with an identifier number and the link between the code and the subject's personal details will only be known by the research team member. Unless the participant gives consent for their personal data to be kept for re-contacting purposes in the event of future research, it will be destroyed.

5.1.3 Where will the data be stored? Who will have access to the data, and what are the data protection measures put in place for this study?

All research data will be stored at the research site under restricted access. Only research team members will have access to the data. The link between the code and the subject's personal details will be kept separately in a secure cabinet or laptop. Documents with identifiers will also be password protected.

5.1.4 The research data will be stored in the following manner:

☒ Coded

☐ Irreversibly de-identified

Who will perform the de-identification (irreversible)/ coding? At which stage of the research will data be irreversibly de-identified/ coded?

The code will be assigned to the participant during informed consent taking by the research team member obtaining consent.

5.1.5 Will individually-identifiable information obtained from the research subject be used for future biomedical research? If so, please ensure you obtain consent.

No, only coded research data will be kept for use in future biomedical research, if the research subject has indicated consent for this in the Informed Consent Form.

5.1.6 What will happen to the research data after research completion? For NUS researchers, please note that under the NUS Research Data Management Policy, research data should be retained for at least 10 years if used in publication and, as an example, research data based on clinical samples or data relating to public health may need to be retained for 15 to 20 years to enable long-term follow up, as might be required.

The research data will be consolidated and stored under a secured facility with the principle investigator for a minimum of 10 years when the study is completed. The records will be accessible for inspection and copying by authorized authorities.

5.1.7 Any other remarks?

None.

☐ *5.2* ***If research involves human tissue****, please complete the following:*

5.2.1 Describe the type of human tissue that will be collected and stored from human research subjects prospectively. Please state the amount and frequency at which these human tissue are taken, and state if trained personnel will obtain the human tissue from the research subjects.

Not Applicable

5.2.2 If you are using archived/existing human tissue, please state the type(s), source(s) and quantity in the table below.

Type of Human Tissue	Source (& Catalog No, if any).	Quantity

In relation to paragraph 5.2.1 and 5.2.2 above, if the human tissue are ***commercially-available****, please confirm the following:*

	YES	NO
a. *No valuable consideration was or will be provided to the tissue donors and/or commercial source(s), apart from providing reimbursement for reasonable costs and expenses incurred in the process of collecting and supplying the human tissue.*	☐	☐
b. *Appropriate consent was obtained from the donors for the human tissue to be used in research, in accordance with the legal or ethical requirements of the country of origin. The intended use of the materials is in accordance with any restrictions and conditions specified by the donors.*	☐	☐

**Please also submit the supplier's declaration confirming the above.*

In relation to paragraph 5.2.1 and 5.2.2 above, if these samples are ***not commercially-available****, please submit the following:*

- *Correspondence to show that the source(s)+ has donated or will be donating the tissue voluntarily;*
- *IRB approval letter and IRB-approved participant information sheet & consent form from an Institutional Review Board which approved the collection of these human tissues.*

+If the human tissue has been obtained from any hospital, please submit approvals from the custodian (e.g., Head of Department) and institutional representative (e.g., Vice Chairman of Medical Board, Research).

5.2.3 What is the specific research purpose for which the human tissue will be used?

5.2.4 *Will the human tissue be used for any purpose other than research, e.g., therapeutic or diagnostic purposes? If so, please state the specific purpose for which the tissue will be used.*

5.2.5 *What clinical data (e.g., age, diagnosis) will be provided with the human tissue?*

5.2.6 *What tests will be performed on these human tissue?*

5.2.7 *Where will the human tissue be stored? What will happen to the human tissue after the research is completed i.e., will they be destroyed, discarded or stored and used for future research?*

5.2.8 *How are the human tissue identified?*

☐ *No Identifiers*

☐ *The materials are coded and the code is maintained with the custodian*

☐ *Identifiers present. Please list the identifiers:* _____

☐ *Others, please specify:* _____

Will the human tissue be used in an individually-identifiable form? If so, please state the reason(s).

5.2.9 *How will the records identifying the donor(s) be kept? To what extent are these records kept confidential?*

5.2.10 *Will the human tissue be used in restricted human biomedical research involving human-animal combinations? If so, please specify the details.*

5.2.11 *Will results from the tests be communicated to the research subjects? If so, how will this be done?*

5.2.12 *Will the human tissue be exported or removed from Singapore to a place outside Singapore? If so, please state the reason(s) and details.*

5.2.13 *Will the human tissue be obtained or was the human tissue obtained from an adult or minor who lacks mental capacity, or from a minor who lacks sufficient understanding and intelligence to give consent?* ☐ *Yes* ☐ *No*

If YES, please confirm the following:

YES ***NO***

a. The human tissue was or will be primarily removed for therapeutic and diagnostic purposes. If YES, please proceed to questions b and c. If NO, please proceed to paragraph 5.2.14 for the waiver application.	☐	☐
b. Appropriate consent has been obtained from their authorised representative for the removal of tissue for the said therapeutic and diagnostic purposes and additional appropriate consent has been obtained for the use of the tissue for research purposes.	☐	☐
c. All necessary therapeutic or diagnostic procedures on these tissue have been completed and they are no longer required for the donor's treatment.	☐	☐

*5.2.14 If you have answered **NO** to paragraph 5.2.13(a) above, please apply for the following waiver:*

☐ *I am applying for a waiver of the requirement that human tissue be removed primarily for therapeutic or diagnostic purposes for (i) adults/minors who lack mental capacity, or (ii) minors who lack sufficient understanding and intelligence to consent.*

In applying for this waiver, I confirm that the following requirements are met:

	YES	***NO***
a. Removal of the human tissue involve no more than minimal risk to the subject.	☐	☐
b. The proposed areas of research cannot be carried out without the use of tissue from the class of persons to which the subject belongs.	☐	☐
c. Appropriate consent has been obtained for use of the human tissues for research by the authorised persons.	☐	☐

5.2.15 Any other remarks?

☐ *5.3 **If research involves human cell cultures/cell lines**, please complete the following:*

Not Applicable

5.3.1 Describe the cell cultures/cell lines that will be used in the research.

Name of Human Cell Lines *(e.g. HeLa)*	Source & Catalog No. *(e.g. ATCC, CCL-2)**	Cell cultures are: *(Please tick)*	
		Primary	Secondary

**For commercially-available cells, please provide proof of purchase or catalog details. For non-commercially available cells, please submit correspondence of willingness from the source(s).*

6. Characteristics of Target Research Subjects (Prospective Recruitment Only):

6.1 *What is the number of research subjects to be enrolled? Give a breakdown by site of recruitment for multi-centred studies.*

Site(s) of Recruitment	Total	Adults (at least 21 years old)	Minors (below 21 years old and never married)
National University of Singapore (Mind Science Center, CHI Study)	100	100	0

6.2 *Lower Age Limit*: 60 *Upper Age Limit:* 85

Total number of research subjects targeted for enrolment worldwide (for international multi-centred studies): Not Applicable

6.3 *Are there any recruitment restrictions based on race or gender of the participant? If yes, please elaborate. If no, please state "Not Applicable".*

Not Applicable

6.4 *Inclusion criteria:*

1. Older adults aged 60 to 85 years
2. Has Mild Cognitive Impairment based on their cognitive diagnosis in the CHI study. These participants were previously diagnosed based on a consensus from a panel of psychiatrists/psychologists who reviewed the Mini-Mental State Examination (MMSE), a Neurocognitive Assessment (NCA) test battery and Clinical Dementia Rating (CDR) scores.
3. Pre-frail as determined by having a score of **1** or **2** on the FRAIL Questionnaire

6.5 *Exclusion criteria:*

1. Engages in vigorous exercises as determined by having more than **0** minutes of vigorous exercise on the International Physical Activity Questionnaire (IPAQ).
2. Has medical contraindications for exercising, including but not limited to: physical disabilities or heart conditions where the primary doctor disallows exercising at moderate intensity.
3. Does not own an Android phone which can support at least a version 6.0 Operating System

6.6 *Are the research subjects vulnerable or in a dependent relationship with the researchers? (e.g. teacher-student relationship, supervisor-staff relationship)*

☐ Yes ☒ No ☐ Not applicable

If Yes, please provide details.

Please note that research subjects who are in a dependent relationship with the researchers should not be approached directly during recruitment, so as to prevent situations where subjects consent under duress.

7. Participant Information Sheet and Written Appropriate Consent:

7.1 *The PI is responsible for ensuring that all research subjects, or persons authorised to give consent on their behalf, give appropriate consent before enrolling into the research. Please submit a copy of the Participant Information Sheet and Consent Form using our template (NUS-IRB-HBRA-GUIDE-03; download latest version from http://www.nus.edu.sg/research/irb/guidelines) for the IRB's review.*

Note:* A Consent Form is NOT required where the research involves solely survey procedures, interview procedures or observation of public behaviour, where data collection is anonymized, *e.g. anonymous surveys. Instead, please apply for a waiver of requirement for appropriate consent to be in writing in Section 7.6.1 and answer all the required questions.*

7.2 *Please explain the proposed procedure for the taking of appropriate consent, having regard to the requirements set out in Section 6 of the HBRA. Please include details of how appropriate consent will be obtained and who will obtain consent.*

The consent process will take place before any initiation of study procedures. An informed consent will be scheduled for interested participants who are eligible for this study. The informed consent will be taken in the presence of an impartial witness in accordance with HBRA, if necessary. The study team member involved in the consent taking process must also state clearly at the beginning that the decision to participate is entirely up to the participant and that he/she can decide not to participate or can withdraw their consent at any time by informing the PI. The study team member must not in any way engage in persuasion or coercion.

A quiet private room will be used to carry out the consent process to minimise disruptions and for privacy. Ample time will be provided to the participant to consider their decision to participate in the research study. This will be done in various ways. The research assistant will go through the clauses in the informed consent form line by line, allowing the participants to stop the researcher at any point to ask questions they might have. The participant will also be given sufficient time to consider or go back and discuss with their family members before agreeing to partake in the research study. A short version of the consent document will be available in Chinese.

7.3 *If minors will be recruited, please specify (a) how you will determine if they have sufficient understanding and intelligence that enables them to understand what is proposed in the proposed research and are capable of giving consent to the proposed research, having regard to the ages, psychological states and maturity of the minors or class of minors involved; and (b) how consent will be obtained from them.*

Not applicable

7.4 *What are the circumstances (if any) under which the research subject or the person authorized to give consent on behalf of the subject will be contacted for further consent?*

For example, changes in the research, serious adverse events that would lead to a change in the research, development of capacity by minors to make decisions.

If there are any changes to the research study, research subjects will be contacted for further consent to these changes.

7.5 *Please fill in this section if you are applying for waiver(s) of the requirement to obtain appropriate consent.*

Not applicable

In applying for any of the waiver(s) below, I acknowledge the following:

☐ *My research does not involve the removal of human tissue from a person.*

☐ *Any waiver(s) granted by the IRB does not affect my duty to protect the individually-identifiable information from unauthorised disclosure under sections 29 and 39 of the HBRA, or such other requirements as may be imposed by law.*

☐ *Any waiver(s) granted by the IRB does not provide immunity to the custodian of any individually-identifiable information for such information if disclosed, unless the disclosure was done in accordance with the HBRA*

7.6 ***I am applying for a waiver of*** *(please check all that apply, and explain how each requirement is fulfilled):*

Not applicable

7.6.1

☐ ***Documentation of Appropriate Consent (i.e., Requirement for Appropriate Consent to be in Writing)***

7.6.1.1 The research or use of the human tissue involves no more than minimal risk to the research subjects or donors ***and*** *involves no procedures for which written consent is ordinarily required outside of a research context (for therapeutic or diagnostic purposes) OR*

7.6.1.2 The only record linking the research subjects to the research or use of the human tissue is the consent form, ***and*** *the principal risk to the research subjects or donors is potential harm resulting from unauthorized disclosure of confidential information such as the research subject's identity and the fact of the subject's participation in the research.*

7.6.2

☐ ***Appropriate Consent (i.e. Requirement for Appropriate Consent for Human Biomedical Research involving Human Biological Material or Health Information) – please tick the sub-category that applies:***

☐ ***For Human Biological Material or Health Information that was obtained after 1 November 2017***

*7.6.2.1 The research cannot reasonably be carried out **without** the use of the human biological material or health information in an individually-identifiable form.*

7.6.2.2 The process of obtaining consent from the person, to which the individually-identifiable human biological material or health information relates, will involve a disproportionate amount of effort and resources relative to the research requirements.

7.6.2.3 The use of the individually-identifiable human biological material or health information involves no more than minimal risk to the research subjects or donors.

7.6.2.4 The waiver will not adversely affect the rights and welfare of the subjects or donors.

7.6.2.5 The research would reasonably be considered to contribute to the greater public good.

☐ ***Individually-identifiable Health Information that was obtained or compiled <u>before</u> 1 November 2017***

*7.6.2.6 The research cannot reasonably be carried out **without** the use of the health information in an individually-identifiable form.*

7.6.2.7 The use of the individually-identifiable health information involves no more than minimal risk to the research subjects.

7.6.2.8 The waiver will not adversely affect the rights and welfare of the subjects.

7.6.2.9 The process of obtaining consent from the person, to which the individually-identifiable health information relates, will involve a disproportionate amount of effort and resources relative to the research requirements.

☐ ***Individually-identifiable Human Biological Material that was obtained or compiled <u>before</u> 1 November 2017***

7.6.2.10 The research cannot reasonably be carried out without the use of human biological material in an individually-identifiable form.

7.6.2.11 Use of the individually-identifiable human biological material involves no more than minimal risk to the research subjects.

7.6.2.12 The waiver will not adversely affect the rights and welfare of the research subjects.

7.6.2.13 Reasonable effort has been made to re-contact the person to which the individually-identifiable human biological material relates for the purpose of obtaining his or her consent.

7.6.3

☐ ***Parental/Legal Guardian Consent (i.e. Requirement for Appropriate Consent to be obtained from Parent/Guardian for Minors to Participate as Research Subjects)***

7.6.3.1 *The research involves no more than minimal risk to the subjects.*

7.6.3.2 *The waiver of parental/legal guardian consent will not adversely affect the rights and welfare of the research subjects.*

7.6.3.3 *The research may not practicably be carried out without the waiver.*

7.6.3.4 *The research proposal is (a) designed for conditions or for a research subject population for which parental or guardian consent is not a reasonable requirement to protect the research subjects (e.g. neglected or abused minors), and an appropriate mechanism for protecting the minors is substituted; or (b) of a private and sensitive nature such that is not reasonable to require permission (e.g. adolescents in studies concerning treatment of sexually transmitted diseases).*

7.6.4

☐ ***Requirement for Appropriate Consent for Emergency Research***

7.6.4.1 *The research subjects are in a life-threatening situation.*

7.6.4.2 *There is no professionally accepted standard of treatment or the available treatments are unproven or are unsatisfactory.*

7.6.4.3 *The collection of valid scientific evidence is necessary to determine the safety and effectiveness of a particular intervention or treatment.*

7.6.4.4 *Participation in the proposed research holds out the prospect of direct benefit to the research subjects.*

7.6.4.5 *It is not feasible to obtain appropriate consent because (a) the subjects will not have capacity within the time available to give their appropriate consent as a result of their medical condition/situation; and (b) no person who is authorized to give appropriate consent on behalf of the research subject is available.*

7.6.4.6 *The research may not practicably be carried out without the waiver.*

7.6.4.7 *Provision is made for* ***one*** *of the following, whichever occurs first:*

	YES	NO
a. *The* ***research subject*** *is to be informed as soon as is practicable after he/she regains capacity of his/her participation in the research and*	☐	☐

given an opportunity to withdraw from further participation in the research; OR

b. The ***person authorized to give appropriate consent on behalf of the research subject*** *is to be informed as soon as is practicable of the subject's participation in the research and to be given an opportunity to request that the subject be withdrawn from further participation in the research;* ☐ ☐

7.6.4.8 *Provision has been made for a* ***medical practitioner*** *who is registered under the Medical Registration Act (Cap. 174) as a specialist in the specialty relating to the research and who is not involved in the research (as a researcher or supervisor) to certify,* ***prior to*** *enrolment of the research subject, that sections 7.6.4.1 – 7.6.4.5 have been complied with to the best of his/her knowledge.*

8. Recruitment Process:

8.1 *Explain the process of recruitment in detail. For example, state how the list of potential research subjects will be obtained e.g. from attending doctor who will refer potential research subjects. Please submit a copy of any recruitment material (e.g. advertisements, recruitment emails) that will be used. You are advised to obtain approval from relevant authorities prior to disseminating any recruitment materials.*

Potential participants are picked from an ongoing elderly cohort study – Community Health and Intergenerational study (CHI study). A member of that research team will contact participants who are eligible and agree to be contacted for future related research. The research team member will ask participants several questions regarding their eligibility and interest over the phone. If they meet the inclusion and exclusion criteria and are interested to participate in the study, they will be invited to attend an informed consent taking session.

A study team member will then take informed consent according to HBRA guidelines.

8.2 *Will research subjects be chosen from medical records? If so, how will you obtain names and NRIC Numbers of research subjects and from whom will you obtain permission for use of these records?*

No

9. Data Safety Monitoring Plan (*for research with more than minimal risk*):

9.1 *Please include details on the Data Safety Monitoring Plan (DSMP) for the research, e.g. the frequency of review and type of data that will be monitored. Please also discuss the plans in place to ensure the safety and well-being of subjects, and integrity of the data collected.*

The team of investigators will perform data and safety monitoring. A review will be conducted every 3 months to monitor for any adverse events that occur that could affect the welfare of the participants or integrity of the results. Data will be evaluated for adherence with the protocol.

In the event that an adverse event is found to have occurred, the PI will collect, record and report to NUS-IRB within the stipulated time frame. Complaints received by any study team member should be reported to the PI within 2 working days.

The PI will be responsible for disseminating any data and safety information to the study team members.

Research assistants involved with data collection will be properly trained and adequately monitored to ensure a good standard of data collection. All data written on the case report forms must not be erased nor thrown away. If needed, cancellations must be accompanied by signatures of the person making the changes. All paper trails must be kept and not thrown away for accounting purposes. Cross-checks of the data collated with the case report forms must be done during the process of the research study to ensure accuracy.

10. Timelines:

10.1 What are the estimated start and end dates of this research? Please note that you should not commence your research prior to IRB approval.

Start Date: August 2020 *End Date:* August 2021

11. Financial Aspects/Conflicts of Interest:

11.1 Who will be responsible for research related costs? For sponsored research, list the costs that will be borne by the sponsor.

This study is funded by The Hong Kong and Shanghai Banking Corporation. It gave a grant for Dementia Prevention and Management Programme in the NUS Mind Science Centre of which this project is a part of. Research related costs will be borne by this fund.

11.2 Will research subjects receive payment/ student course credits for participation? If yes, please elaborate. If no, please state "No reimbursement".

Subjects will receive reimbursement for their time, transportation costs and inconvenience caused from their participation in this study. They will receive $10 for each visit. In total, they will receive $30 upon completion of their participation in this study.
